# Sulforaphane counteracts aggressiveness of pancreatic cancer driven by dysregulated Cx43-mediated gap junctional intercellular communication

**DOI:** 10.18632/oncotarget.1764

**Published:** 2014-01-22

**Authors:** Tobias Forster, Vanessa Rausch, Yiyao Zhang, Orkhan Isayev, Katharina Heilmann, Frank Schoensiegel, Li Liu, Michelle Nessling, Karsten Richter, Sabrina Labsch, Clifford C. Nwaeburu, Juergen Mattern, Jury Gladkich, Nathalia Giese, Jens Werner, Peter Schemmer, Wolfgang Gross, Martha M. Gebhard, Clarissa Gerhauser, Michael Schaefer, Ingrid Herr

**Affiliations:** ^1^ General, Visceral and Transplantation Surgery, University of Heidelberg, Heidelberg, Germany; ^2^ Experimental Surgery, University of Heidelberg, Heidelberg, Germany; ^3^ Epigenomics and Cancer Risk Factors, German Cancer Research Center (DKFZ), Heidelberg, Germany; ^4^ Core Facility Electron Microscopy, German Cancer Research Center (DKFZ), Heidelberg, Germany

**Keywords:** Cancer Stem Cells, Pancreatic Cancer, Bioactive dietary agents, Sulforaphane

## Abstract

The extreme aggressiveness of pancreatic ductal adenocarcinoma (PDA) has been associated with blocked gap junctional intercellular communication (GJIC) and the presence of cancer stem cells (CSCs). We examined whether disturbed GJIC is responsible for a CSC phenotype in established and primary cancer cells and patient tissue of PDA using interdisciplinary methods based in physiology, cell and molecular biology, histology and epigenetics. Flux of fluorescent dyes and gemcitabine through gap junctions (GJs) was intact in less aggressive cells but not in highly malignant cells with morphological dysfunctional GJs. Among several connexins, only Cx43 was expressed on the cell surface of less aggressive and GJIC-competent cells, whereas Cx43 surface expression was absent in highly malignant, E-cadherin-negative and GJIC-incompetent cells. The levels of total Cx43 protein and Cx43 phosphorylated at Ser368 and Ser279/282 were high in normal tissue but low to absent in malignant tissue. si-RNA-mediated inhibition of Cx43 expression in GJIC-competent cells prevented GJIC and induced colony formation and the expression of stem cell-related factors. The bioactive substance sulforaphane enhanced Cx43 and E-cadherin levels, inhibited the CSC markers c-Met and CD133, improved the functional morphology of GJs and enhanced GJIC. Sulforaphane altered the phosphorylation of several kinases and their substrates and inhibition of GSK3, JNK and PKC prevented sulforaphane-induced CX43 expression. The sulforaphane-mediated expression of Cx43 was not correlated with enhanced Cx43 RNA expression, acetylated histone binding and Cx43 promoter de-methylation, suggesting that posttranslational phosphorylation is the dominant regulatory mechanism. Together, the absence of Cx43 prevents GJIC and enhances aggressiveness, whereas sulforaphane counteracts this process, and our findings highlight dietary co-treatment as a viable treatment option for PDA.

## INTRODUCTION

Pancreatic ductal adenocarcinoma (PDA) is one of the most aggressive malignancies and is typically diagnosed in an advanced state [1]. Gemcitabine is the standard therapy for advanced pancreatic cancer [2] but neither gemcitabine nor the newer FOLFIRINOX combination therapy do not directly target cancer stem cell (CSC) pathways responsible for growth, metastasis, high resistance toward current cancer therapeutics and relapse [3-5].

The mustard oil sulforaphane, which is present in high concentrations in broccoli and its sprouts [6], has well-documented cancer preventive efficacy and was recently shown to inhibit histone deacetylases (HDACs) [7]. Our recent results demonstrated that sulforaphane sensitizes pancreatic CSCs to chemotherapy by inhibiting their self-renewal potential, apoptosis resistance and NF-κB activity [8-11]. Based on several promising animal and epidemiological studies, prospective clinical trials with sulforaphane-enriched broccoli sprout extracts are ongoing in the U.S. to examine the effect on atypical nevi, bladder and prostate cancer [12], and a pilot study recently began at our clinic to evaluate the effect of this nutritional strategy on patients with advanced PDA.

The inhibition of gap junctional intercellular communication (GJIC) may be involved in the autonomous behavior of CSCs, because GJIC confers features absent in stem cells, namely contact inhibition [13], apoptosis sensitivity [14] and terminal differentiation [15]. Gap junctions (GJs) are channels in the plasma membrane that allow direct communication between cells by the passive transport of small molecules and ions [16]. Gemcitabine and other chemotherapeutics also diffuse from cell-to-cell via GJs [17], and this so-called “bystander effect” leads to a higher cellular concentration of cytotoxic agents [18]. GJs are formed by a family of more than 20 different connexins, and altered connexin expression is involved in carcinogenesis and therapy resistance [19]. The absence of GJs is a common feature of pluripotent stem cells. However, cells derived from stem cells, e.g., progenitor cells, have functional GJs, suggesting that GJIC develops during differentiation [20-22]. Recently, in human spheroidal glioma cultures, reduced GJIC and very low levels of Cx43 were identified as mediators of self-renewal, invasiveness, and tumorigenicity by influencing E-cadherin expression, a marker of epithelial-mesenchymal transition (EMT) [23]. The phosphorylation of Cx43, the most ubiquitously expressed connexin, has been implicated in the regulation of GJIC at several stages, e.g., the export of the protein to the plasma membrane, the formation and activity of GJs and connexin degradation [24]. For example, the inactivation of ERK1/2 and p38 MAP kinases by sulforaphane has been suggested to inhibit H_2_O_2_-induced phosphorylation of Cx43 and a block of GJIC in rat normal liver cells [25]. In addition, epigenetic mechanisms may be involved in the regulation of Cx43 protein levels, because previous findings demonstrated that the HDAC inhibitor 4-phenylbutyrate increased the expression of Cx43 in pancreatic cancer cells and promoted the growth inhibition of xenograft tumors [26]. Similarly, the hypermethylation of the Cx43 promoter was correlated with low Cx43 expression in human gliomas and lung cancer [23, 27].

In the present study, we demonstrate that the degree of CSC features and gemcitabine resistance directly correlates with dysfunctional GJIC due to low or absent Cx43 protein levels. Our results provide new mechanistic insight into stem cell signaling and suggest a dietary strategy consisting of sulforaphane-containing cruciferous vegetables for the restoration of defective GJIC in pancreatic cancer, which could promote re-sensitization to current chemotherapeutics.

## RESULTS

### The loss of GJIC correlates with a CSC-like phenotype and gemcitabine resistance

**Figure 1:**
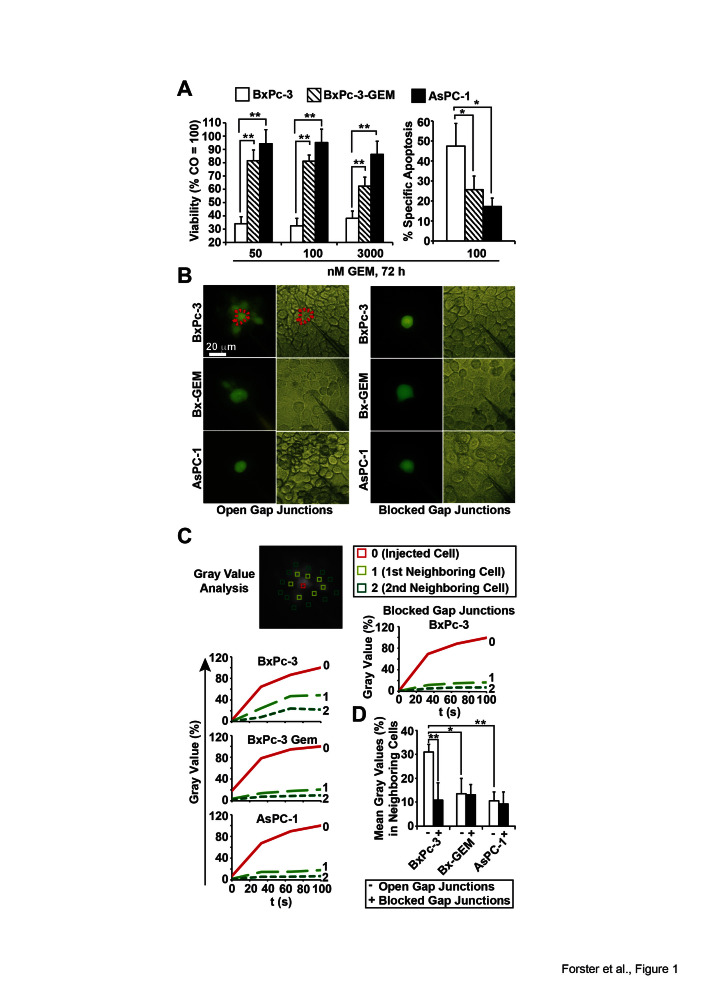
Loss of GJIC correlates with a CSC-phenotype. (A) BxPc-3, BxPc-3-GEM and AsPC-1 human PDA cells were treated with gemcitabine (GEM) at the indicated concentrations. Seventy-two hours later, viability was measured with the MTT assay and apoptosis by annexin staining followed by FACS analysis. Specific apoptosis was calculated using the formula 100× [(experimental apoptosis %) - spontaneous apoptosis of CO (%)] / [100 - spontaneous apoptosis of CO %]. (B) After microinjection of Lucifer Yellow the diffusion of dye from the injected cell to neighboring cells was detected by fluorescence microscopy and video recording in the presence or absence of the gap junction blocker 18αGA (10 mM), which was incubated for 30 min prior to the injection of Lucifer Yellow. Representative images from fluorescence and light microscopy are shown. Representative cells injected with Lucifer Yellow are marked by dotted lines, and the scale bar indicates 20 μm. (C) Gray values of the injected cell (0, red line), the first raw of neighboring cells (1, light green-dotted line) and the second raw of neighboring cells (2, middle green-dotted line) were determined from the video pictures at the time points 0, 20, 40, 60, 80 and 100 s after injection of lucifer yellow and are shown in the diagrams. (D) The means of gray values of all neighboring cells per cell line were calculated and are shown in the diagram ± SD. **p < 0.01; *p< 0.05.

To evaluate the relationship between GJIC and therapy resistance, 3 established human PDA cell lines with different levels of therapy sensitivity were treated with 50, 100 or 3,000 nM gemcitabine and cell viability was examined using the MTT assay 72 h after treatment. At the lowest dose, cell viability was reduced from 100% to 34, 82 and 95% in sensitive BxPc-3, the derived gemcitabine-resistant subclone BxPc-3-GEM and resistant AsPC-1 cells, respectively, with a comparable response at higher gemcitabine concentrations (Fig. 1A). This result was confirmed by measuring apoptosis with annexin staining and FACS analysis (Fig. 1A). The gemcitabine resistance of the PDA cell lines correlated with the amount of CSC features, such as p53 and K-ras status, morphology, self-renewal potential, tumorigenicity, and the expression of E-cadherin and vimentin (Table S1). In the following experiments, these cells were used as *in vitro* models for PDA with low (BxPc-3), median (BxPc-3-GEM) and high (AsPC-1) CSC characteristics. We microinjected the membrane-impermeable but GJ-permeable fluorescent dye Lucifer Yellow [30] and documented diffusion of fluorescence to neighboring cells by fluorescence microscopy and video recording. For data analysis gray values of fluorescence intensity were evaluated by image processing and the gray value of the directly injected cell was set to 100% (Fig. 1B, C). The gray values of direct neighboring cells in the first row surrounding the injected cell were 50, 20 and 0% in BxPc3, BxPc-3-GEM and AsPC-1 cells, respectively. The staining of indirect neighbors located in the second row was detectable in BxPc-3 cells only. This result is reflected by the evaluation of the means of gray values of all neighboring cells in each cell line, which was highest in BxPc-3 cells (Fig. 1D). The blockade of GJs with 18αGA was used as negative control and completely prevented the diffusion of Lucifer Yellow in all cell lines as expected (Fig. 1C, D). These observations were strengthened by co-incubation studies with fluorescence-labeled cells followed by examination of the fluorescence intensity in unlabeled target cells and by co-incubation of gemcitabine-treated and -untreated cells and studying the gemcitabine bystander effect (Fig. S1).

**Figure 2:**
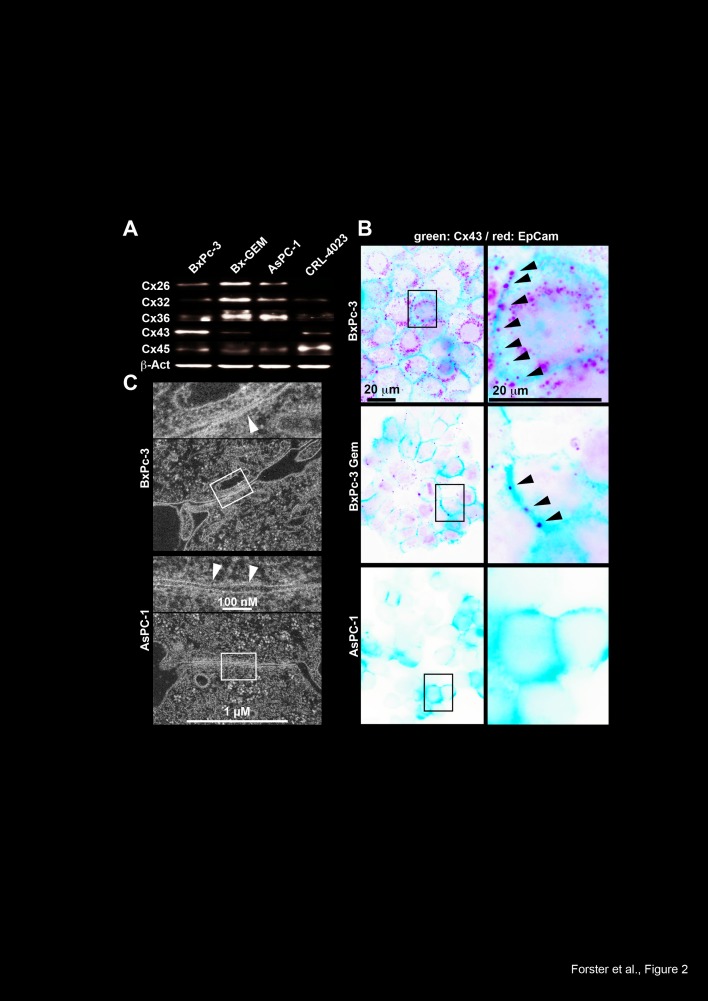
Loss of Cx43 expression in cells with CSC features. (A) Proteins were harvested from PDA cells and immortalized pancreatic ductal cells (CRL-4023), and the expression of Cx26, Cx32, Cx36, Cx43 (anti-Cx43 Ab was from Invitrogen) and Cx45 was analyzed by Western blot. The expression of β-Actin (β-Act) served as a loading control. (B) The expression and cellular localization of Cx43 (Cell Signaling) was analyzed by double immunofluorescence staining using antibodies specific for Cx43. Co-staining with the cell surface protein EpCAM was performed to mark the cell surface. Cx43-positive cells were visualized with Alexa Fluor 488 (green), and EpCAM-positive cells were visualized with Alexa Fluor 594 (red). The arrows mark Cx43 expression on the cell surface. Cells were analyzed under 400× magnification using a Leica DMRB fluorescence microscope. Images of representative fields were captured using a Kappa CF 20/4 DX digital color camera (Kappa GmbH, Gleichen, Germany) and Kappa ImageBase 2.2 software. All photographs were taken at the same magnification, and the scale bar indicates 20 μm. (C) Electron microscopy of gap junction-like cell-cell contacts of BxPc-3 and AsPC-1 cells. The arrows indicate the extension of junctions with a narrow gap in BxPc-3 cells, and the inserts show details of the gaps at higher magnification. The scale bar indicates 1 μm.

To evaluate whether the reduced expression of a specific connexin is responsible for impaired GJIC, we studied the expression patterns of the standard connexins Cx32, 26, 36, 45 and 43 by Western blot analysis. While Cx26, 32 and 36 levels were enhanced in BxPc-3-GEM and AsPC-1 cells compared to BxPc-3 and non-malignant, immortalized pancreatic ductal CRL-4023 cells, Cx43 and 45 levels were diminished in the more malignant cells, with the strongest effects observed for Cx43 compared to BxPc-3 and CRL-4023 cells (Fig. 2A). Because the expression of connexins on the cell surface is essential for GJ functionality, we evaluated the cell surface expression by double immunofluorescence stainings for the cell surface marker EpCAM combined with either Cx26, 32, 36, 43 or 45. In line with the Western blot results, fluorescence microscopy revealed strong expression of Cx43 on the cell surface of BxPc-3 cells; however, this expression was diminished in BxPc-3-GEM and totally absent in AsPC-1 cells (Fig. 2B). The staining patterns of all other connexins were diffuse in all cell lines without obvious cell surface expression (Fig. S2), which argues against a major role in conferring functional GJs. In addition, electron microscopy revealed the presence of functional GJs in BxPc-3 cells, as closely apposed membranes separated by a faint gap were visible (Fig. 2C). In contrast, gap-like structures between neighboring AsPC-1 cells were characterized by constant spacing and gaps filled with contrasting material. These gaps were too wide for regular GJ arrays; therefore, our data suggest that non-functional GJs are present in AsPC-1 cells, which is most likely due to the absence of Cx43 on the cell surface.

**Figure 3:**
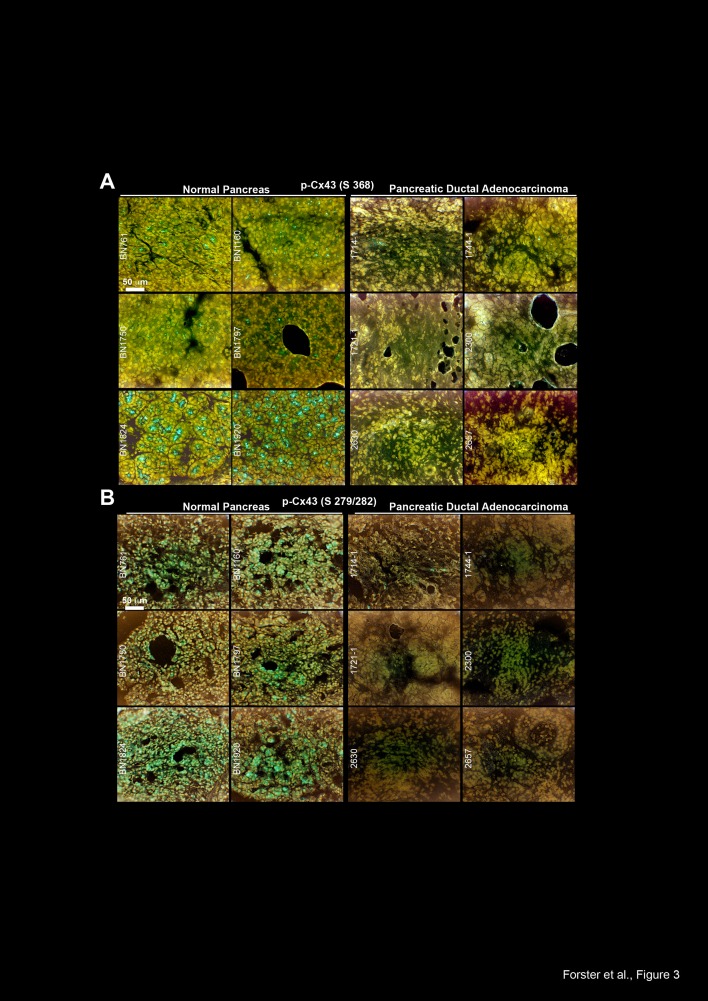
Cx43 is downregulated in malignant pancreas tissue but not in normal pancreas tissue (A) The expression of Cx43 phosphorylated at Ser 368 (Ab from Abcam) was analyzed by immunohistochemistry in frozen 6-μm-thick tissue sections of non-malignant human pancreatic tissue derived from 6 organ donors. Labeled anti-rabbit polymer-HRP was used as the secondary antibody. (B) Likewise, the expression of Cx43 phosphorylated at Ser 279/282 was analyzed in the same tissues and by the same technique.

**Figure 4:**
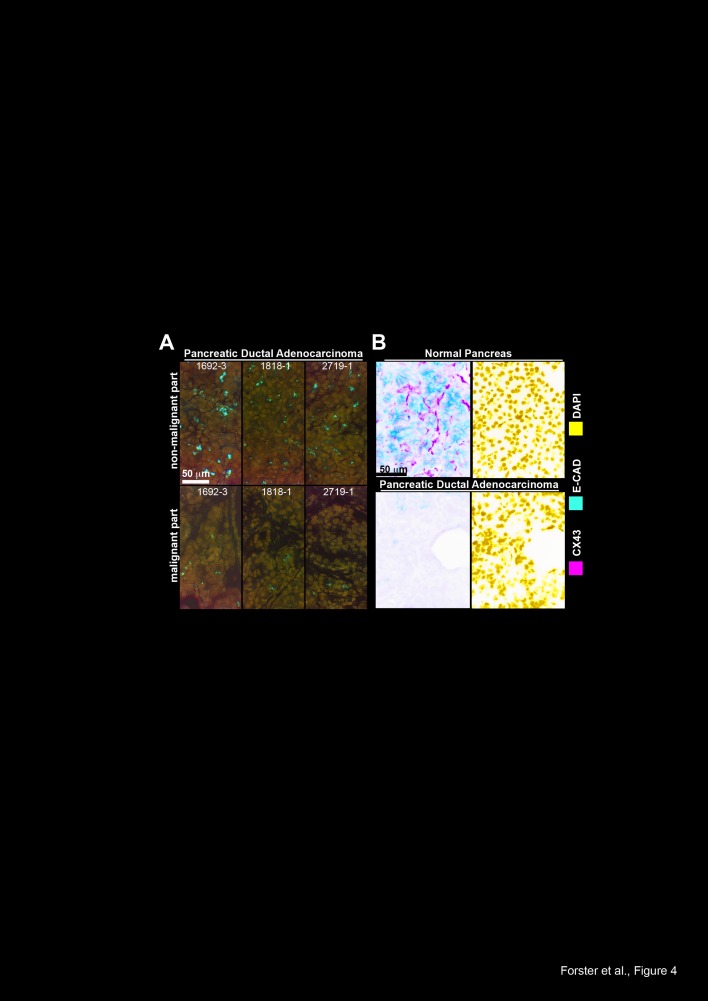
Cx43 and E-Cadherin are downregulated in malignant but not in non-malignant parts of pancreas tissue from the same patients (A) The expression of total Cx43 (Invitrogen) was analyzed in the malignant and non-malignant parts of frozen 6-μm-thick tissue sections of pancreas tissue derived from 3 patients. (B) The expression of total Cx43 (Invitrogen) and E-cadherin (E-CAD) was analyzed by double immunofluorescence staining of malignant and non-malignant parts of a patient-derived PDA tissue section. Cx43-positive cells were visualized with Alexa Fluor 488 (green), and E-cadherin-positive cells were visualized with Alexa Fluor 594 (red). DAPI staining indicates the cell nuclei. Tissue sections were analyzed under 400× magnification. The scale bars indicate 50 μm.

In surgically resected human pancreas tissue we found high levels of Cx43 phosphorylated at Ser 368 and at Ser 279/282 in non-malignant pancreas tissue derived from 6 organ donors but not in malignant PDA tissue derived from 6 patients (Fig. 3A, B). These data were confirmed in additional PDA tissues derived from 3 patients, in which total Cx43 was completely absent in the malignant parts of the sections but present in the non-malignant parts of each individual tissue (Fig. 4A). This correlated to expression of E-cadherin, which was expressed in normal pancreas tissue, but not in malignant pancreas tissue, as examined by double immunofluorescence staining of total Cx43 and E-Cadherin followed by fluorescence microscopy (Fig. 4B).

### The inhibition of Cx43 prevents GJIC and promotes tumor aggressiveness

**Figure 5:**
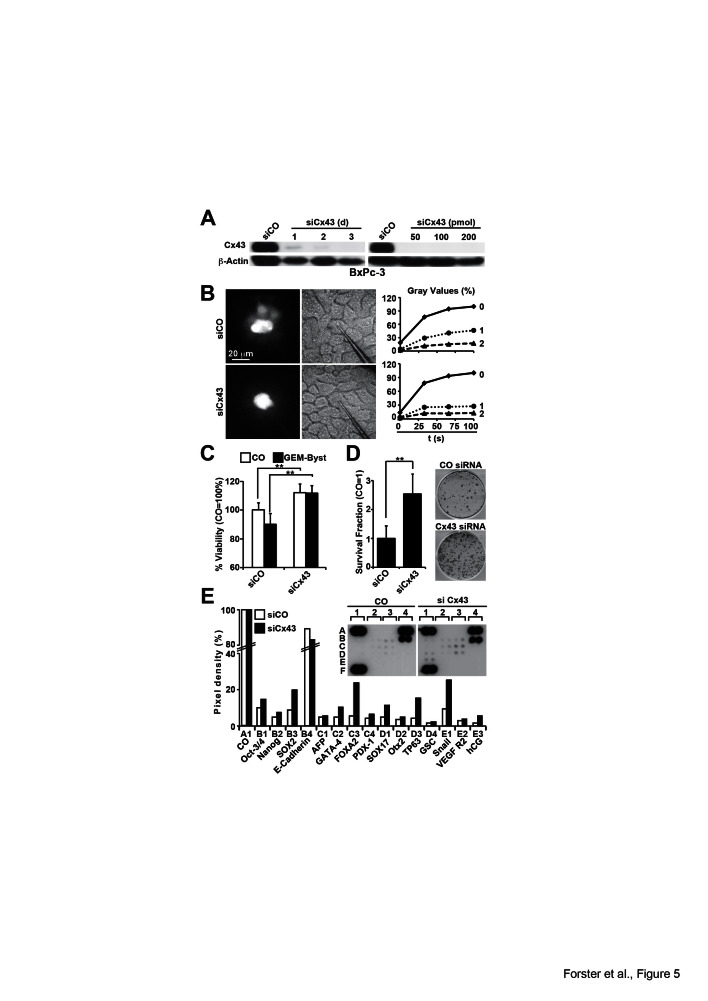
Silencing of Cx43 blocks GJIC and induces therapy resistance and clonogenicity (A) BxPc-3 cells were treated with non-specific siRNAs (siCO) or specific siRNAs directed against Cx43 (siCx43) for 3 days. The protein expression of Cx43 was analyzed by Western blot on days 1, 2 and 3 after incubation with siRNA or 3 d after incubation with siRNA at concentrations of 50, 100 or 200 pmol, as described in Fig. 3A. (B) BxPc-3 cells were treated with 50 pmol siRNA for 72 h followed by microinjection of Lucifer Yellow, and the monitoring of dye diffusion was performed as described in Fig. 1C. Pictures were extracted from the videos at distinct time points, and the gray values were calculated. The average gray value of the first neighbors (1) and second neighbors (2) and the gray value of the injected cell (0) are shown as the percentage over a period of 100 s. The data shown represent the means of 3 independent experiments. (C) The cells were treated with 50 pmol siRNA against Cx43 (siCx43) or non-specific control siRNA (siCO) for 72 h. Then, the cells were left untreated or were treated with gemcitabine for 24 h, followed by co-culture at a ratio of 1:1 for 24 h. The viability of untreated cells (CO, white bars) and of cells co-cultured with gemcitabine-treated cells (GEM-Byst, black bars) was measured using the MTT assay. (D) Three days after transfection with siCO or siCx43, 1×10^3^ BxPc-3-GEM cells/well were seeded onto 6-well plates. The cells were grown without a change of the medium for 2 weeks, followed by the evaluation of fixed and Coomassie-stained colonies containing at least 50 cells. The percentage of plating efficiency was calculated with the formula 100× number of colonies/number of seeded cells. The data shown represent the means ± SD (**p<0.01). (E) Proteins were isolated 3 d after transfection of BxPc-3 cells with siCO or siCx43, and the binding of proteins to antibodies spotted in duplicate to the membrane of a Human Pluripotent Stem Cell Array was detected using biotinylated secondary antibodies, streptavidin-HRP and chemiluminescence. The pixel density was quantified using ImageJ software and normalized to the mean pixel intensity of reference spots located at the coordinates A1, A4 and F1 on the membrane. Spot E4 is the negative control, where PBS instead of the antibody was spotted onto the membrane. The mean values from duplicate experiments with a similar outcome are shown.

To further elucidate the function of Cx43 for GJIC and CSC features in pancreatic cancer, we knocked down Cx43 in low-malignant BxPc-3 cells by Cx43-directed siRNAs. Compared to the non-specific control siRNA, 2 of 5 tested specific siRNAs completely downregulated Cx43 protein expression at a concentration of 50 pmol, and the strongest effects were observed 3 days after transfection, as evaluated by Western blot analysis (Fig. 5A, Fig. S3). For subsequent experiments, siRNA-transfected BxPc-3 cells were microinjected with Lucifer Yellow, followed by video analysis of dye diffusion and analysis of gray values. Compared to cells transfected with control siRNA, the specific siRNA targeting Cx43 decreased the average staining of the neighboring cells in the first cell row from 50 to 30% and in the second row from 20 to 15% (Fig. 5B). In parallel, the viability of Cx43 siRNA-transfected BxPc-3 cells increased and the gemcitabine bystander effect was completely abolished, as measured by the co-incubation of gemcitabine-treated and non-treated cells (Fig. 5C). Furthermore, the downregulation of Cx43 enhanced the colony-forming capacity (Fig. 5D) and led to the induction of stem cell-associated factors including Oct-3/4, Nanog, SOX2 and TP65 as well as the EMT-related transcription factor Snail, as measured using an antibody protein array (Fig. 5E).

### Sulforaphane enhances Cx43 protein levels and GJIC and changes Cx43 phosphorylation patterns

**Figure 6:**
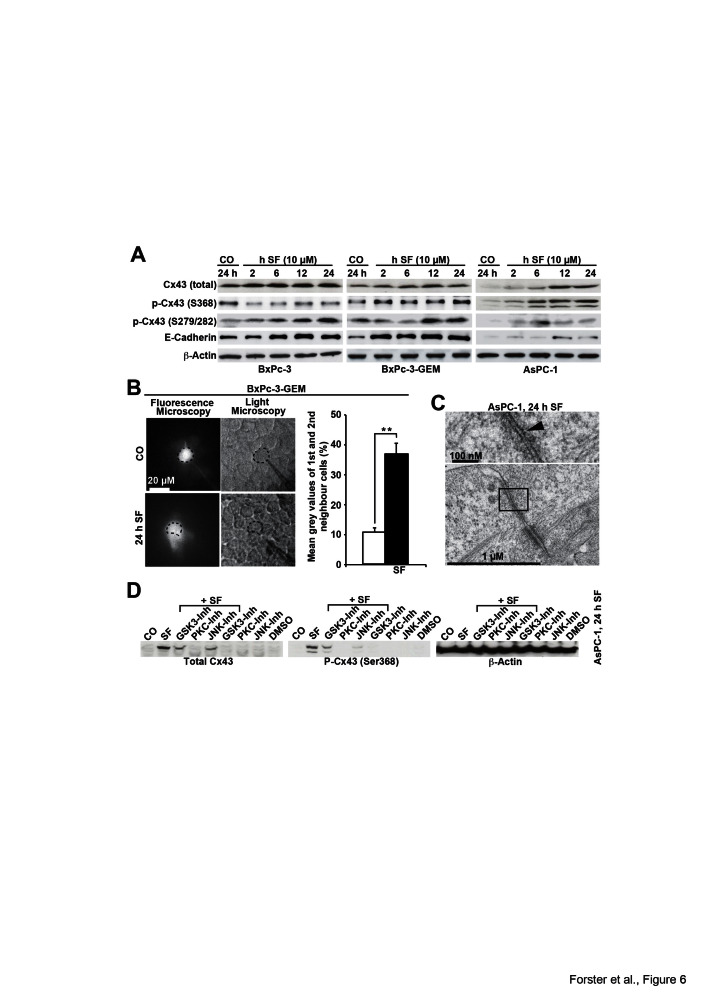
Sulforaphane enhances GJIC and Cx43 protein expression, which is prevented by inhibition of kinase activity (A) BxPc-3, BxPc-3-GEM and AsPC-1 cells were left untreated or were treated with sulforaphane (SF, 10 μM) and time points indicated. The proteins of all treatment groups were harvested at the same time point, and Western blot analysis was performed using antibodies to detect total Cx43 with an antibody, which does not detect Cx43 phosphorylated at Ser 368 (Invitrogen), Cx43 phosphorylated at Ser 368 (Abcam), Cx43 phosphorylated at Ser 279/282, E-cadherin and β-Actin. (B) BxPc-3 cells were treated with sulforaphane (SF, 10 μM) or were left untreated (CO). Twenty-four hours later, the cells were analyzed as described in Fig. 5B. The mean gray values of the 1^st^ and 2^nd^ neighbors of all time points are shown. (C) Electron microscopy of AsPC-1 cells, which were left either untreated or were treated with sulforaphane (SF, 10 μM) for 24 h, was performed as described in Fig. C. (D) AsPC-1 cells were left untreated or were treated with sulforaphane (SF, 10 μM) in the presence or absence of the GSK3-inhibitor BIO (10 μM), the PKC-inhibitor staurosporine (100 nM) or the JNK-inhibitor SP600125 (10 μM). Treatment with the inhibitors alone or with the solvent DMSO served as control. Proteins were harvested 24 h after treatment and the expression of total Cx43, Cx43 phosphorylated at Ser 368 and β-Actin was examined by Western blot analysis.

In an attempt to enhance Cx43 expression to restore GJIC and to inhibit CSC features, we performed treatments with sulforaphane, and examined the expression of Cx43 by Western blot analysis. Compared to untreated control cells, sulforaphane strongly increased the amount of total Cx43 in all cell lines examined (Fig. 6A). Basal Cx43 phosphorylated at Ser 368 was highest in BxPc-3 cells. Sulforaphane led to a time-dependent increase of the low basal levels of Cx43 phosphorylated at Ser 368 already 2 h after treatment in BxPc-3-GEM and AsPC-1 cells, whereas the high basal expression in BxPc-3 cells was not further increased, but rater decreased. Moreover, the expression of Cx43 phosphorylated at Ser 279/282 was increased by sulforaphane treatment in all 3 cell lines. Sulforaphane also induced the expression of E-cadherin in all 3 cell lines, but stronger expression was observed in the 2 more aggressive cell lines demonstrating lower basal levels. To evaluate whether sulforaphane-mediated induction of Cx43 expression was accompanied by enhanced GJIC, we microinjected Lucifer Yellow into untreated or sulforaphane-treated BxPc-3-GEM cells. Video analysis of dye diffusion and the analysis of gray values revealed that sulforaphane increased GJIC (Fig. 6B). The results of electron microscopy correlated with these findings, as the contrasting material in gap-like structures between neighboring AsPC-1 cells was diminished after sulforaphane treatment (Fig. 6C) compared to untreated AsPC-1 cells (Fig. 2C). Finally, inhibition of GSK3, PKC and JNK by the inhibitors BIO, staurosporine and SP600125, respectively, diminished the sulforaphane-induced expression of total Cx43 and of Cx43 phosphorylated at Ser 368 as shown in AsPC-1 cells (Fig. 6D). This result, together with the finding that sulforaphane influences the phosphorylation of numerous kinases and their substrates in a time-dependent manner (Fig. S4) suggests that postranslation phosphorylation is involved in enhanced expression of Cx43. In contrast, sulforaphane did not upregulate Cx43 mRNA expression, as measured by qRT-PCR (Fig. S5); did not increase the demethylation of the Cx43 promoter, as measured by quantitative methylation analysis of the *GJA1* (Cx43) gene (Fig. S6); and did not enhance the binding of acetylated histones to the *GJA1* promoter as measured by chromatin immunoprecipitation of acetylated histones 3 and 4 followed by the analysis of several regions surrounding the *GJA1* transcription start site by qPCR (Fig. S7). Remarkably, enhanced methylation of the Cx43 promoter in untreated AsPC-1 cells compared to BxPc-3 and BxPc-3-GEM cells was detected (Fig. S6), which may be responsible for the non-detectable expression of Cx43 mRNA in AsPC-1 cells (Fig. S5). Therefore, elevated Cx43 protein levels after sulforaphane were most likely due to the altered phosphorylation and stabilization of Cx43 protein; although the phosphorylation pattern of Cx43 varied between cell lines, which is in line with a recent report [31].

**Figure 7:**
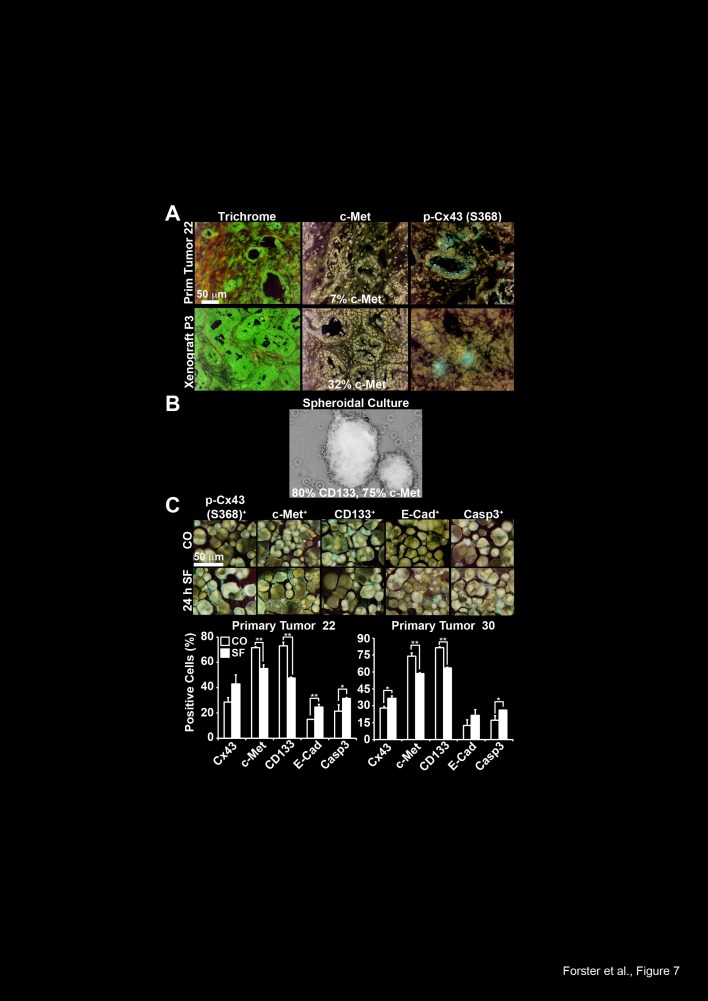
Sulforaphane induces Cx43 and inhibits CSC characteristics in primary CSCs (A) Staining of patient-derived frozen tissue from a ductal adenocarcinoma (Primary Tumor 22) and it´s derived xenograft from passage 3 (Xenograft P3) with Trichrome, c-Met or Cx43 phosphorylated at Ser 368 (Abcam), followed by microscopical evaluation under 400× magnification. The scale bar indicates 50 μm. (B) Representative picture of an anchorage-independent growing spheroidal culture established from a mouse xenograft derived from the primary patient tumor 22. The percentage of expression of the CSC markers c-Met and CD133 was determined as described in part C and is indicated. (C) One week after *in vitro* spheroidal culture, cells derived from pancreatic ductal adenocarcinoma 22 and 30 were left untreated or were treated with sulforaphane (10 μM). Twenty-four hours later, the cells were cytospinned to glass slides, and the expression of Cx43 phosphorylated at Ser 368 (Abcam), c-Met, CD133, E-cadherin and the cleaved, active fragment of Caspase-3 was examined by immunohistochemistry. The number of positive cells was quantified in 10 vision fields under 400× magnification and the means ± SD are shown in the diagrams. **p<0.01, *p<0.05.

To further highlight these findings in primary CSC-marker-positive cells, we selected primary CSCs by transplantation of freshly resected PDA tissue from two patients to immunodeficient mice. For enrichment of CSCs we subtransplanted the xenografts for several rounds. During subtransplantation, the tumor morphology and tumor stroma were largely preserved, as ensured by Trichrome staining of the primary tumors and its xenografts (Fig. 7A and data not shown), whereas the latency and the tumor take rate increased (data not shown). Accordingly, the CSC marker c-Met was enriched from 17% in the primary tumor to 32% in xenografts at passage 3. The expression of Cx43 phosphorylated at Ser 368 was also detected in the primary tumor tissue and its xenografts; however, quantitative evaluation was not possible due to the expression of Cx43 between neighboring cells. To examine the effect of sulforaphane on CSC features, we further enriched CSC-marker positive cells from passage 7 xenografts of the 2 patient tumors by isolation of tumor cells and *in vitro* spheroidal culture (Fig. 7B). This culture technique favored the growth of CSCs, because the ability to grow anchorage-independent is considered as a property of CSCs, and this is reflected by the enhanced expression of the CSC markers c-Met (~ 75%) and CD133 (~ 80%), as detected by the preparation of cytospins and immunohistochemistry (Fig. 7C). Moreover, the treatment of the spheroidal cultures with sulforaphane for 24 h increased the expression level of Cx43 phosphorylated at Ser 368, E-cadherin and active caspase 3, whereas the levels of c-Met and CD133 decreased. These results demonstrate that Cx43 expression is low in primary CSCs, but can be increased by sulforaphane treatment, which is associated with inhibition of CSC and EMT markers and induction of apoptosis.

## DISCUSSION

We examined GJIC in human pancreatic cells and tissues and found a direct correlation among low Cx43 protein levels, blocked GJIC, gemcitabine resistance, CSC features and EMT marker expression. In particular, less malignant and GJIC-competent BxPc-3 cells expressed Cx43 protein at a high level on the cell surface and showed morphologically intact GJs; however, Cx43 was not expressed in highly malignant AsPC-1 cells with blocked GJIC and morphologically non-functional GJs. BxPc-3-GEM cells were intermediate malignant subclones of BxPc-3 cells and exhibited weaker expression of Cx43, which was primarily localized to the cytoplasm rather than to the cell surface. These findings suggest that cytoplasmic localization of Cx43 induces the dysregulation of GJs by Cx43 withdrawal from the cell membrane during early tumor progression (BxPc-3-GEM), which may be followed by complete silencing of Cx43 expression in later tumor stages (AsPC-1). In this regard, changes in Cx43 expression and localization during pancreatic cancer progression have been recently described in the murine system and suggest a potential role for GJs and Cx43 in mediating interactions between and amongst the stromal and epithelial cells [32]. Another suggested mechanism for Cx43 translocation from the cell surface, along with the reduction of GJIC, involves the hypophosphorylation of Cx43, as demonstrated in oncogene-transformed rat liver epithelial cells and the occurrence of phosphorylated (P_1_, P_2_ P_2_´_,_ P_3_) and unphosphorylated P_0_ Cx43 immunoreactive bands in Western blot analysis [33]. This hypothesis corresponds to our stainings of human pancreatic cancer tissues, in which the high expression of Cx43 phosphorylated at Ser 368 and Ser 279/282 was detected in non-malignant tissue only. The key phosphorylation events at different Cx43 serine residues responsible for regulating the GJ life cycle are complex and differentially regulated between different cell types and amino acid residues, and were therefore not examined in detailed in the present report. However, the underlying signaling pathways of Cx43 phosphorylation appear to involve MAPK activity [31]. In this regard, the activity of JNK, ERK and p38 MAP kinases and the subsequent enhanced phosphorylation of Cx43 have been shown to inhibit Cx43 protein expression [25, 34]. In our studies, the sulforaphane-mediated elevated levels of Cx43 were prevented by inhibitors of GSK3, JNK and PKC, which suggests the involvement of these kinases in phosphorylation of Cx43, which may lead to enhanced protein levels. This suggestion is underlined by our finding that sulforaphane influenced the phosphorylation patterns of several kinases and their substrates in a time-dependent manner.

To demonstrate the strong impact of Cx43 on GJIC in pancreatic cancer, we inhibited Cx43 expression by siRNA transfection in GJIC-competent BxPc-3 cells, leading to a completely abrogated GJIC, which was associated with a higher basal viability, resistance to gemcitabine, enhanced clonogenic potential and the induction of stem cell-associated factors. This finding is supported by observations made by Yu and colleagues [23], who identified the expression of Cx43 in more differentiated and GJIC-competent human glioma cells, while glioma stem cells were GJIC-incompetent and Cx43 negative. Hence, adenoviral reconstitution of Cx43 expression in glioma stem cells inhibited their capacity for self-renewal, invasiveness and tumorigenicity by influencing E-cadherin expression [23]. As an underlying mechanism for reduced Cx43 expression in glioma stem cells, these previous authors suggested that hypermethylation of the promoter of the GJ protein α1, which encodes Cx43 [23], may regulate this process. This hypothesis was evaluated by measuring the immunoprecipitation of methylated DNA (MeDIP) followed by PCR amplification of 2 potentially methylated regions in the *GJA1* (Cx43) promoter. We confirmed this result in our system and used quantitative EpiTYPER methylation profiling of the region surrounding the *Cx43* transcription start site. We also detected enhanced *Cx43* promoter methylation in CSC-like AsPC-1 cells compared to the less malignant BxPc-3 and BxPc-3-GEM cells. Thus, strongly enhanced promoter methylation of Cx43 may be responsible for the observed absent RNA and protein expression of Cx43 in AsPC-1 cells. However, it remains unclear how dysfunctional GJIC supports a CSC phenotype and tumor progression. A reasonable explanation is that dysfunctional GJIC prevents influences from surrounding normal tissue cells [35], thereby enabling CSCs to escape growth control mechanisms and apoptosis induction [20, 36].

We demonstrated that sulforaphane stimulated the elevated expression of defective Cx43 in established and primary models of pancreatic CSCs along with the restoration of defective GJIC, the activation of E-cadherin protein and the inhibition of the CSC markers CD133 and c-Met. A main underlying mechanism for these observations may be postranscriptional phosphorylation, because inhibition of GSK3, JNK and PCS inhibited the sulforaphane-induced expression of Cx43 and sulforaphane influenced the phosphorylation of several kinases and their substrates. We did not observe an effect of sulforaphane on Cx43 RNA expression, promoter methylation or promoter binding of acetylated histones; therefore, the epigenetic regulation of Cx43 expression by sulforaphane may be excluded. However, our data do not exclude the possibility that sulforaphane generally affects epigenetic regulation, as demonstrated by our Western blot experiments showing enhanced acetylation of histones 3 and 4 after sulforaphane treatment of PDA cells. In addition, work by others has demonstrated the inhibition of DNA methyltransferase and histone deacetylase by sulforaphane [37, 38]. Our results also do not exclude the involvement of histone acetylation in the basal regulation of Cx43 expression in pancreatic cancer, which has been suggested by a recent study showing that the HDAC inhibitor 4-phenylbutyrate increases Cx43 expression and suppresses the growth of human pancreatic cancer cells [26].

The present data demonstrate that reduced Cx43 protein levels are major mediators of blocked GJIC, which confer a CSC-like phenotype in PDA. However, dietary sulforaphane enhances Cx43 protein levels, restores GJIC and inhibits CSC features; therefore, a diet rich in cruciferous vegetables containing sulforaphane and related mustard oils may be considered as a supportive nutritional strategy to enhance therapeutic efficacy in patients suffering from pancreatic cancer.

## MATERIALS AND METHODS

### Established Cell Lines

Established human BxPc-3 and AsPC-1 PDA cell lines and immortalized CRL-4023 hTERT-HPNE human PDA cells were obtained from ATCC. BxPc-3-GEM subclones were selected from parental BxPc-3 cells by continuous culturing in increasing concentrations of gemcitabine up to 100 nM for several months. Cells were cultured in DMEM (PAA, Pasching; Austria) supplemented with 10% heat-inactivated FCS (Sigma-Aldrich, Steinheim; Germany) and 25 mmol/L HEPES (PAA, Pasching; Austria). Cells were authenticated by a commercial service (Multiplexion, Heidelberg, Germany). Monthly tests ensured a mycoplasma-free cell culture.

### Reagents

A 126 mM gemcitabine solution (Eli Lilly, Indianapolis, IN, USA) used for patients was diluted in cell culture medium to a 100 μM stock solution. The gap junction inhibitor 18α-glycyrrhetinic acid (18GA; Sigma-Aldrich, St. Louis, MO; USA) [39] was diluted in DMSO to a 70 mM stock. D,L-sulforaphane (>90% pure, Sigma-Aldrich, Steinheim; Germany) was dissolved to create a 50 mM stock solution in EtOH. 5-Aza-2'-deoxycytidine (>97% pure, Sigma) was dissolved in acetic acid:water (1:1) to create a 50 ng/ml stock solution. The GSK-3 inhibitor BIO and the JNK-inhibitor SP600125 (Selleckchem.com, München, Germany) were dissolved in DMSO to 10 mM stocks and the PKC-inhibitor staurosporine (Selleckchem.com) was dissolved in DMSO to a 1 mM stock. The final concentrations of the solvents in medium were 0.1% or less.

### Primary Antibodies

Rabbit polyclonal antibodies (pAbs) were used against Cx32, Cx26 (Invitrogen, Camarillo, California; USA), c-Met, total Cx43 (#3512, Cell Signaling Technology, Boston, MA, USA), Cx43 phosphorylated at Ser 368 (#ab47368; Abcam, Cambridge, UK), Cx45 phosphorylated at Ser 279/282 (Santa Cruz Biotechnology, Inc. Heidelberg, Germany), Cx45, CD44 (GeneTex, Irvine, California, USA), acetyl-Histone H3 and acetyl-Histone H4 (Merck Millipore, Darmstadt, Germany), E-cadherin (Cell Signaling), Ki67 (Thermo Scientific, Rockford, IL, USA) and the cleaved fragment of activated human caspase-3 (R&D Systems, Abingdon, UK). Mouse monoclonal antibodies (mAbs) were used against Cx36, total Cx43 (which does not detect Cx43 phosphorylated at Ser 368, #138300; Invitrogen, Camarillo, California; USA), CD133 (Abcam), β-Actin (Sigma, St. Louis, MO, USA), EpCAM and the epithelial glycoprotein Egp34 (kindly provided by G. Moldenhauer and described [40]). Another rabbit monoclonal antibody (mAb) was used to detect Cx43 phosphorylated at Ser 368 (#GTX48551 (Gene Tex (Irvine, California, USA).

### Primary Spheroidal Cells

Surgical non-diagnostic specimens were mechanically minced, and 2×10^7^ cells placed in Matrigel were transplanted to the flanks of 6-week-old NMRI (nu/nu) female mice. The tumor take rate was 60%, and after the development of a tumor, the xenografts were resected, minced and subtransplanted to new mice. Subtransplantation was repeated until stably growing xenograft lines after passage 3 were obtained. Pancreatic cancer spheres were generated from 2 established xenograft lines, as recently described [4, 41], and the primary spheroidal cultures were used for experiments between days 7 and 30 of culture. Patient material was obtained under the approval of the ethical committee of the University of Heidelberg after written informed consent was obtained from patients. Diagnoses were established by conventional clinical and histological criteria according to the World Health Organization (WHO). All surgical resections were indicated according to the principles and practice of oncological therapy. Animal experiments were also approved by the ethical committee.

### Microinjection of Lucifer Yellow

The tip of an ultrathin self-made glass pipette was positioned in the intracellular space of a single cell in a subconfluent cell layer in 35-mm dishes. The position was controlled by measuring the membrane potential via the wire in the pipette and the ground electrode in the culture medium. Lucifer Yellow dye (Sigma Aldrich, Germany) was injected with a customized iontophoresis device for 90 s at a constant current of 10 nA. The distribution of Lucifer Yellow was examined by fluorescence microscopy (Zeiss, Oberkochen; Germany) in combination with a CCD Kappa CF 15 DRE camera (Kappa GmbH, Gleichen, Germany) and a video recorder, as recently described [42]. Pictures were extracted from the videos between the onset (t = 0 s) and the end of the injection (t = 100 s). The fluorescence signal was analyzed at 100 s by calculating the gray values of the directly injected cell and the 1^st^ and 2^nd^ cellular neighbors using customized image data processing software (Histo 3.0, University Hospital Heidelberg, Germany). The gray value of the directly injected cells was set to 100%, and the gray value of the 10^th^ neighboring cell was set to 0%. To block GJIC, the cells were treated with 10 μM 18α-glycyrrhetinic acid (18αGA; Sigma-Aldrich, St. Louis, MO; USA) 30 min prior to microinjection.

### Electron Microscopy

Cells were processed for electron microscopy according to standard procedures. Briefly, the 3-step fixation process included 1 h on ice in 2.5% glutaraldehyde (EM-grade, Sigma) buffered to pH 7.2 with 40 mM Na-cacodylate, 2.4 mM MgCl_2_, 50 mM KCl and 58 mM sucrose, followed by 1 h on ice in 1% aqueous OsO_4_ and overnight at 4°C in 0.5% aqueous uranyl acetate. After dehydration in graded steps of ethanol, the adherent cells were flat-embedded in epoxy resin (Polysciences, Eppelheim, Germany). Ultrathin sections parallel to the substrate-plane were prepared at a 40-nm nominal thickness, post-stained in Reynold's lead citrate and 4% aqueous uranyl acetate and viewed with an EM900 transmission electron microscope (Zeiss, Oberkochen, Germany) at 80 kV. Images were captured on image plates at 16,000× primary magnification and were scanned with a 15-μm step-size (Ditabis Micron, Pforzheim, Germany).

### Antibody Protein Array

Nitrocellulose membranes, on which capture antibodies were spotted, and reagents for detection were obtained as a kit (Human pluripotent stem cell antibody array, Human phospho-kinase array) from R&D Systems®(Wiesbaden, Germany). According to the manufacturer's instructions, protein extracts were prepared and then incubated with the nitrocellulose membranes, and specific protein binding was detected with biotinylated secondary antibodies using streptavidin-HRP and chemiluminescence detection reagents.

### Protein Isolation and Western Blot Analysis

Whole-cell extracts were prepared using a standard protocol, and proteins were detected by Western blot analysis using specific primary antibodies as described above. Goat anti-rabbit or goat anti-mouse secondary antibodies conjugated to horseradish peroxidase (HRP) (Santa Cruz, CA; USA) and Luminata Forte Western HRP Substrate (Millipore Corporation, Billerica, MA; USA) were used for detection.

### MTT Assay

Cells were seeded in 96-well plates at 4×10^3^ cells/well and treated with gemcitabine 24 h later. After an additional 72 h, viability was determined using the 3-(4,5-dimethylthiazol-2-yl)-2,5-diphenyltetrazolium bromide (MTT) assay, as previously described [8].

### Annexin Staining and FACS Analysis of Apoptosis

Apoptosis was detected by annexin staining and flow cytometry as previously described [43] using a BD LSR II flow cytometer and FACS Diva software (BD Biosciences, San Jose, CA; USA). Specific apoptosis was evaluated with the following formula: 100× [(experimental apoptosis %) - (spontaneous apoptosis of CO %)] / [100 - spontaneous apoptosis of CO %].

### FACS Analysis of GJIC

FACS analysis of GJIC was performed as recently described [30]. In brief, 3×10^5^ cells/well in a 6-well plate were labeled for 30 min at 37°C with 3 μM Calcein-AM (Acetoxymethyl ester; Merck, Darmstadt; Germany) or 5 μM CellTracker Red CMTPX (Invitrogen, Eugene, Oregon; USA) diluted in Opti-MEM medium (Invitrogen). Calcein-loaded donor cells were trypsinized and plated on top of the CellTracker Red-stained acceptor cells at a ratio of 1:10. After 6 h of co-incubation, cells were trypsinized, and single- and double-fluorescent cells were evaluated by FACS analysis.

### Colony Forming Assay

Three days after siRNA transfection, 1×10^3^ BxPc-3-GEM cells/well were seeded into 6-well tissue culture plates (BD Falcon™, San José, CA, USA), and colony forming assays were performed as previously described [44].

### The Gemcitabine Bystander Effect

Cells were cultured in T75 flasks at concentrations of 4 to 6×10^6^ cells per flask. Confluent cells were left untreated or were treated with 3 μm gemcitabine for 24 h. Treated cells were mixed at a ratio of 1:1 with untreated cells and were seeded in 6-well plates at a concentration of 7×10^5^ cells/ml. Twenty-four hours later, the cells were trypsinized and seeded at a concentration of 4×10^4^ cells/ml. Next, at 24 or 72 h after co-cultivation, viability was measured using the MTT assay. Untreated cells were used as a control and set to 100%. To measure GJIC by flow cytometry, untreated cells were stained with CellTracker Red CMTPX (Invitrogen, Eugene, Oregon; USA), co-cultured and subjected to annexin staining and FACS analysis of gated CellTracker Red-labeled cells only.

### Immunohistochemistry of Established Cell Lines

Immunofluorescence staining was performed in established cell lines growing in chambers of the Nunc® Lab-Tek Chamber Slide™ system (Sigma-Aldrich). According to a standard protocol, 95% EtOH served as a fixative, and 0.2% Triton X-100 was used for permeabilization [45]. Nuclei were stained with DAPI (4,6-diamidino-2'-phenylindol, 1 μg/ml). The binding of primary antibodies was detected using the following secondary Abs: goat anti-rabbit Alexa Fluor 488 IgG, goat anti-rabbit Alexa Fluor 594 IgG, goat anti-mouse Alexa Fluor 594 IgG and goat anti-mouse Alexa Fluor 488 IgG (Invitrogen, Camarillo, CA; USA). Omission of the primary Ab served as a negative control. The signal was detected using a Leica DMRB fluorescence microscope (Leica, Wetzlar; Germany). Images of representative fields were captured using a SPOT™ FLEX 15.2 64 Mp shifting pixel digital color camera (Diagnostic, Instruments, Inc. USA) and analyzed with SPOT Basic/Advanced 4.6 software.

### Immunohistochemistry of Primary Tissue and Primary Cells

Frozen 6-μm tissue sections from normal and malignant human pancreas tissue obtained from healthy organ donors, patients with PDA, primary mouse xenografts or spheroidal cultures cytospinned to glass slides were fixed with 4% paraformaldehyde. Peroxidase activity was blocked with 0.03% hydrogen peroxide. Immunohistochemistry was performed using EnVision+System-HRP (AEC) (DakoCytomation, Carpinteria, CA; USA). After staining with primary antibodies, the signal was detected as described above for the established cell lines. The omission of the primary Abs was used as a negative control. Patient material was obtained under the approval of the ethical committee of the University of Heidelberg. Diagnoses were established by conventional clinical and histological criteria according to the World Health Organization (WHO). All surgical resections were indicated according to the principles and practice of oncological therapy.

### siRNA Transfection

siRNA against Cx43 (Hs_GJA1_5) was obtained from Qiagen (Maryland; USA), and Mission siRNA Universal Negative Control was purchased from Sigma-Aldrich (St. Louis, MO; USA). Transfections were performed using the Lipofectamine™ RNAiMax kit from Invitrogen (Carlsbad, CA; USA) according to the manufacturer's instructions.

### Statistical Analysis

Data obtained with established and primary cell lines, with the exception of antibody protein arrays, are presented as the mean ± SD of at least three separate experiments, performed in triplicates. Statistical significance was evaluated by Student's t-test with Bonferroni correction (*p<0.05, **p<0.01). Antibody protein arrays were performed twice in duplicates with similar outcome and the means of two experiments are shown.

## SUPPLEMENTARY FIGURES AND TABLES


