# Supplementary material for: Sulforaphane counteracts aggressiveness of pancreatic cancer driven by dysregulated Cx43-mediated gap junctional intercellular communication
Source: Oncotarget. 2014 Jan 22;5(6):1621–34. doi: 10.18632/oncotarget.1764 (PMC4039235; doi:10.18632/oncotarget.1764)
Supplement: Supplementary file 1 [file oncotarget-05-1621-s001.pdf]

SUPPLEMENTAL MATERIAL

SUPPLEMENTARY FIGURE LEGENDS

Figure S1. The impaired gemcitabine bystander effect in therapy-resistant CSC-like cells.

Cells with open (-) or blocked (+) GJs after treatment with 70 μM 18αGA were left untreated or were treated with gemcitabine (3000 nM) for 24 h followed by co-culturing with treated and untreated cells at a ratio of 1:10. Four days later, viability was measured using the MTT assay. The viability of non-gemcitabine-treated cells was set to 100%, and the corresponding percentage of the viability of co-incubated cells is shown.

Figure S2. The evaluation of connexin expression in PDA cell lines.

The expression and localization of connexins was analyzed by immunofluorescence staining of Cx26, Cx32, Cx36 and Cx45 (green) and the co-staining of EpCAM on the cell surface (red). The scale bar indicates 20 μm.

Figure S3. siRNA transfection blocks Cx43 protein expression.

BxPc-3 cells were treated with non-specific siRNA (CO) or 2 different specific siRNAs directed against Cx43 (siCx43 2, siCx43 5) at the indicated concentrations. The concentration of the control siRNA was 100 pmol. Three days later, the protein expression of Cx43 was analyzed by Western blot analysis. The expression of β-Actin served as the loading control.

Figure S4. Sulforaphane influences the phosphorylation of several kinases and their substrates.

AsPC1 cells were left untreated (CO) or were treated with sulforaphane (SF) for 6 and 16 h. Thereafter, proteins were isolated and the binding of phosphorylated proteins to antibodies spotted in duplicate to the membrane of the Human Phospho-Kinase Array Kit was examined The expression of phosphorylated proteins was detected using biotinylated secondary antibodies, streptavidin-HRP and chemiluminescence. The pixel density was quantified using ImageJ software and normalized to the mean pixel intensity of reference spots. The mean values from duplicate experiments with a similar outcome ± SD are shown.

Figure S5. Sulforaphane does not enhance Cx43 mRNA expression.

The RNA expression levels of *Cx43, E-Cad* and *CDKN1a* in BxPc-3, BxPC-3-GEM and AsPC-1 cells were determined by qRT-PCR and normalized to the housekeeping genes (HKG) *HPRT1* and *TBP.* Depicted is the mean of duplicate measurements relative to the untreated control at time 0 h. The expression of Cx43 in AsPC-1 was below the detection limit.

Figure S6. Sulforaphane does not change Cx43 DNA methylation patterns.

(A) A schematic representation of the *GJA1* (Cx43) gene (blue), locations of the EpiTYPER Massarray amplicons (A1-A8) covering the region approximately 1.4 kb upstream and 3 kb downstream of the transcription start site and the location of the amplicons used for the detection of ChIPed DNA (1-5) covering approximately 880 bp upstream and 2.8 kb downstream of the transcription start site is shown. (B) Quantitative EpiTYPER methylation profiling of the region surrounding the *Cx43* transcription start site in the 3 pancreatic cancer cell lines BxPc-3, BxPc-3-GEM and AsPC1. Each row represents a single sample (CO = control; SF = treated with sulforaphane at indicated doses and times; 5Aza = treated with 5-Aza-2'-deoxycytidine at indicated dose and time; STD = 6-point methylation standard from 0% - 100% methylation), and each column depicts a CpG unit (single CpG or group of several CpGs). The degree of methylation is indicated by a color gradient from light yellow (0% methylated) to dark blue (100% methylated). The gray color indicates the missing value.

Figure S7. Sulforaphane decreases the binding of acetylated histones to the *Cx43* promoter. (A) Global acetylation levels of histone H3 and H4 (ac-H3, ac–H4) were analyzed by Western blot in whole-cell lysates of BxPC-3, BxPc-3-GEM and AsPC-1 cells treated with 10 µM sulforaphane (SF) for the indicated times or left untreated for 24 h. β-Actin was used as a loading control. (B, C) Chromatin immunoprecipitation against acH3 (B) or acH4 (C) was performed with BxPc-3 cells treated for 6 h with either solvent control (CO) or varying doses of sulforaphane. The enrichment relative to input was examined by qPCR for several regions surrounding the *GJA1* (Cx43) transcription start site (see Suppl. Fig. 6A) as well as the control region in the *CDKN1a* promoter. Depicted is the mean of duplicate measurements.

Supplementary Table S1. CSC features of established human PDA cell lines

**CSC-<br>Properties**	**Parental<br>BxPc-3**	**Selected<br>BxPc-3-GEM**	**AsPC-1**	**References**
Source	Primary tumor	BxPc-3	Ascites	ATCC
Differentiation primary tumor	Well	Well	Moderate-poor	ATCC
p53 status	MT	MT	MT	[28]
K-ras status	WT	WT	MT	[28]
In vitro morphology	Densely attached	Densely attached	Loosely attached	Present study
Colony-forming capacity	+	++	+++	[8], UOD
ALDH1 activity	-	+	++	[8]
Tumorigenicity in mice	+	++	+++	[8], UOD
$CD44^{+}/CD24^{-}$	< 20%	> 40%	> 90%	[8]
Gemcitabine resistance	+	++	+++	compare Fig. 1A
E-Cadherin protein expression	+++	++	+	[9, 29], compare Fig. 6A
Vimentin protein expression	-	+	+	[9, 29]

None (-); Weak (+); Median (++); Strong (+++); Unpublished Own Data (UOD); MT (mutant), WT (wild-type).

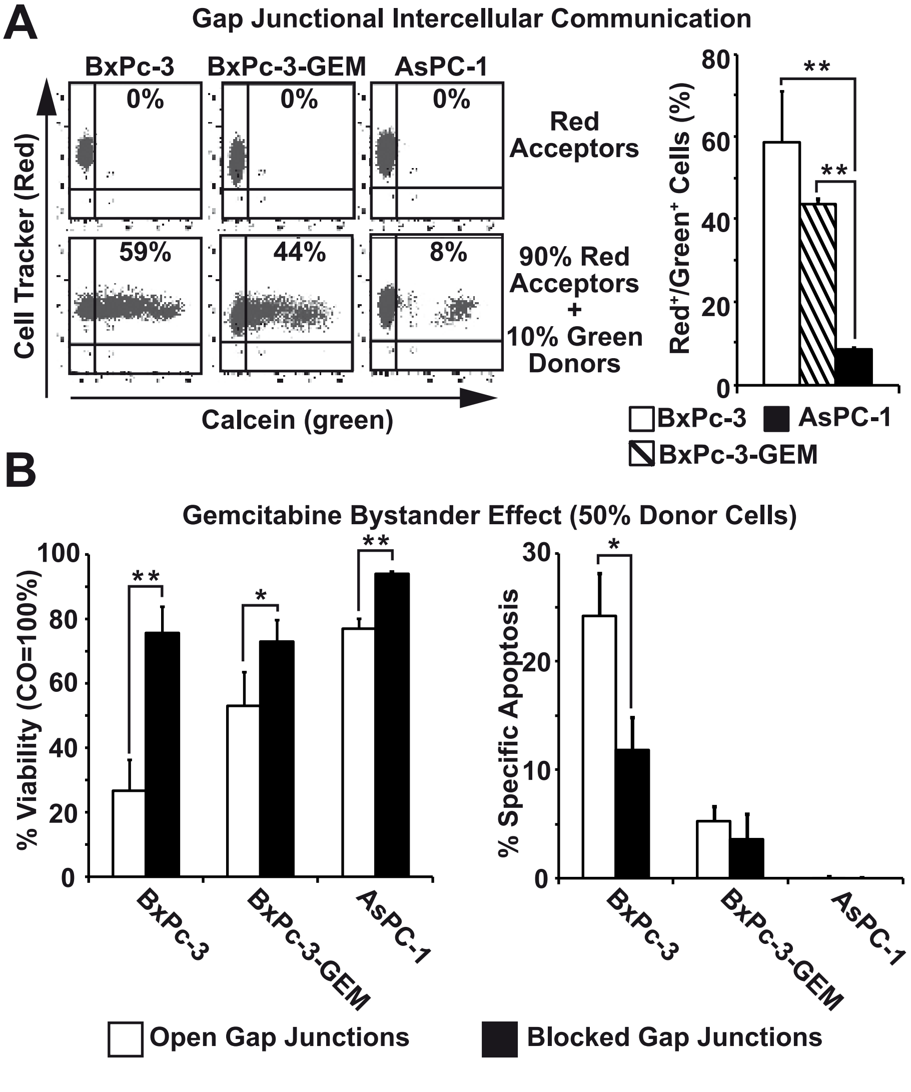
A
Gap Junctional Intercellular Communication
BxPc-3
BxPc-3-GEM
AsPC-1
0%
0%
0%
Red Acceptors
59%
44%
8%
90% Red Acceptors + 10% Green Donors
Cell Tracker (Red)
Calcein (green)
Red+/Green+ Cells (%)
80
60
40
20
0
**
**
BxPc-3
AsPC-1
BxPc-3-GEM
B
Gemcitabine Bystander Effect (50% Donor Cells)
% Viability (CO=100%)
100
80
60
40
20
0
**
*
**
BxPc-3
BxPc-3-GEM
AsPC-1
% Specific Apoptosis
30
20
10
0
*
BxPc-3
BxPc-3-GEM
AsPC-1
Open Gap Junctions
Blocked Gap Junctions

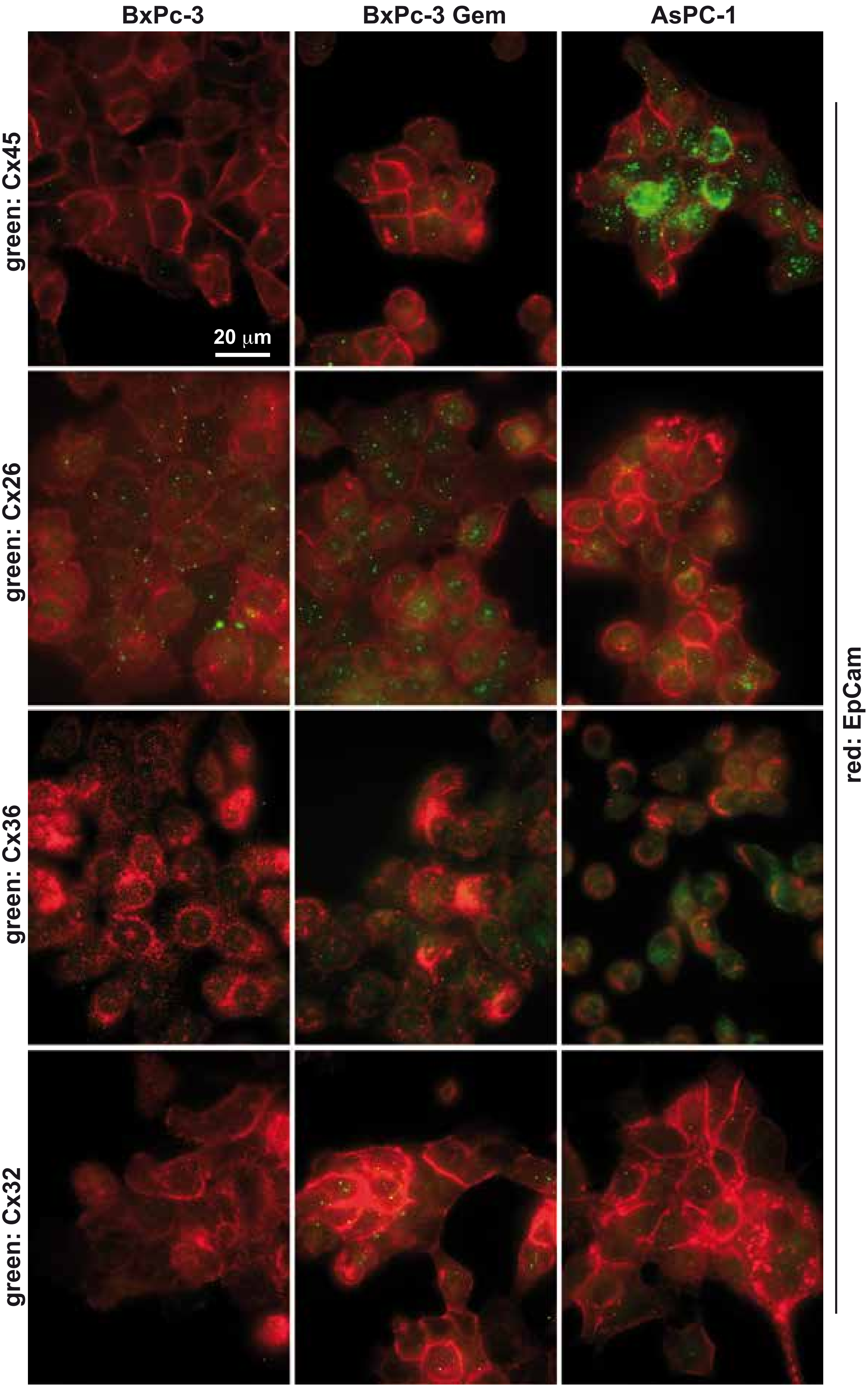

Forster et al., Supplemental Figure 2

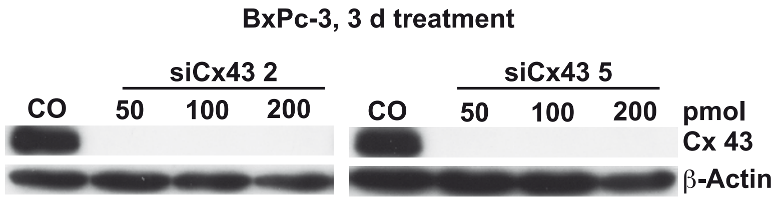

Forster et al., Supplemental Figure 3

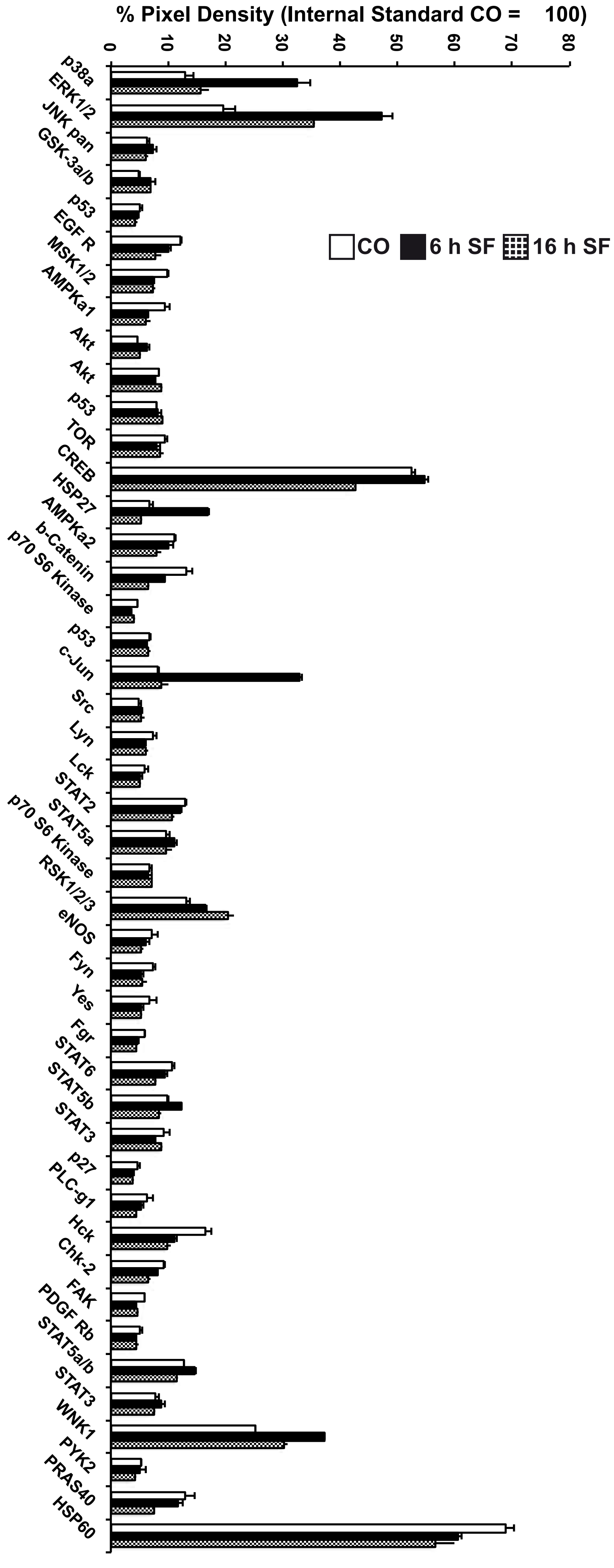
% Pixel Density (Internal Standard CO = 100)
0
10
20
30
40
50
60
70
80
CO
6 h SF
16 h SF
p38a
ERK1/2
JNK pan
GSK-3a/b
p53
EGF R
MSK1/2
AMPKa1
Akt
Akt
p53
TOR
CREB
HSP27
AMPKa2
b-Catenin
p70 S6 Kinase
p53
c-Jun
Src
Lyn
Lck
STAT2
STAT5a
p70 S6 Kinase
RSK1/2/3
eNOS
Fyn
Yes
Fgr
STAT6
STAT5b
STAT3
p27
PLC-g1
Hck
Chk-2
FAK
PDGF Rb
STAT5a/b
STAT3
WNK1
PYK2
PRAS40
HSP60

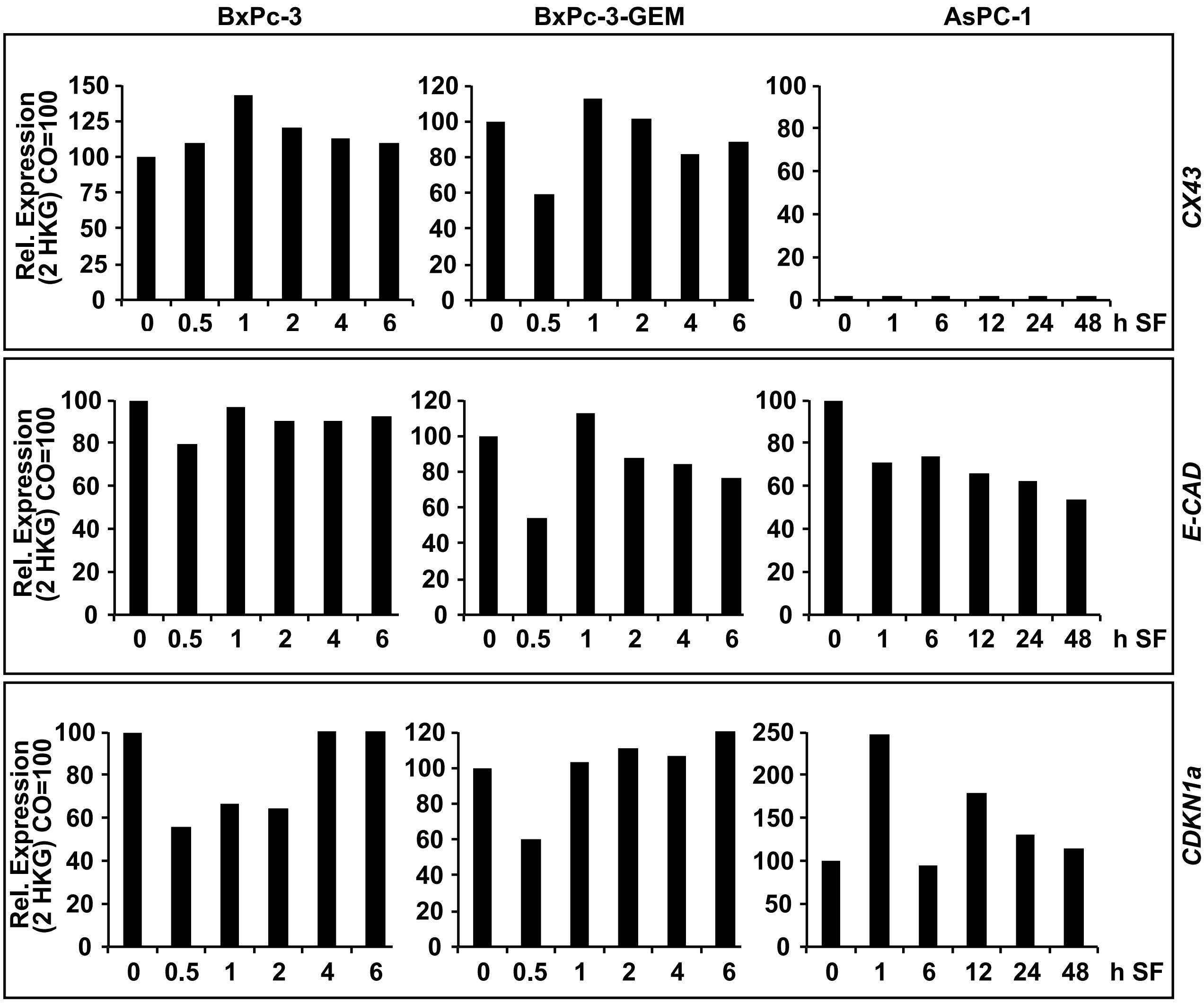

Forster et al., Supplemental Figure 5

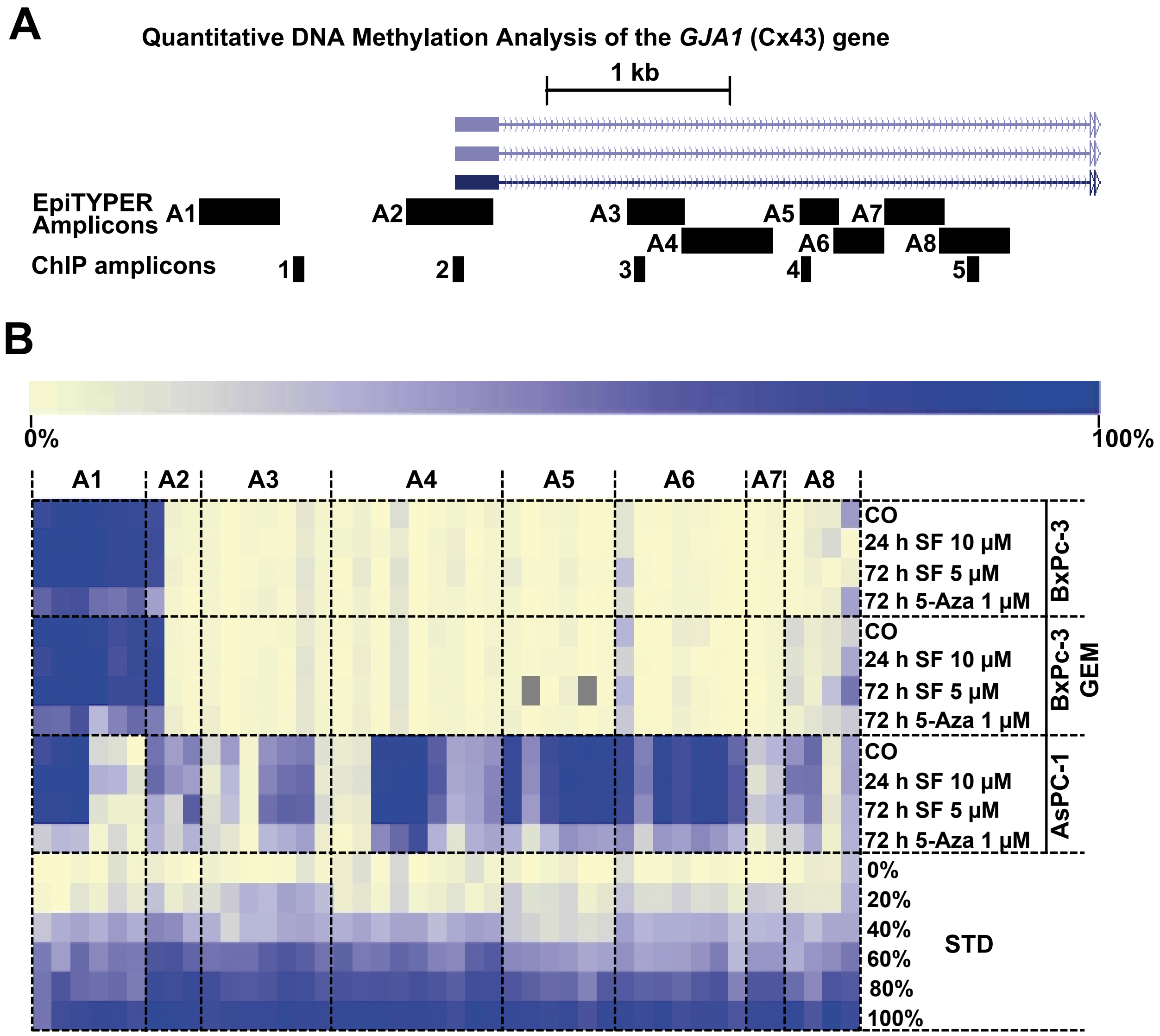
A
Quantitative DNA Methylation Analysis of the *GJA1* (Cx43) gene
1 kb
EpiTYPER Amplicons
A1
A2
A3
A4
A5
A6
A7
A8
ChIP amplicons
1
2
3
4
5
B
0%
100%
A1 A2 A3 A4 A5 A6 A7 A8
CO
24 h SF 10 µM
72 h SF 5 µM
72 h 5-Aza 1 µM
BxPc-3
BxPc-3 GEM
AsPC-1
0%
20%
40%
60%
80%
100%
STD

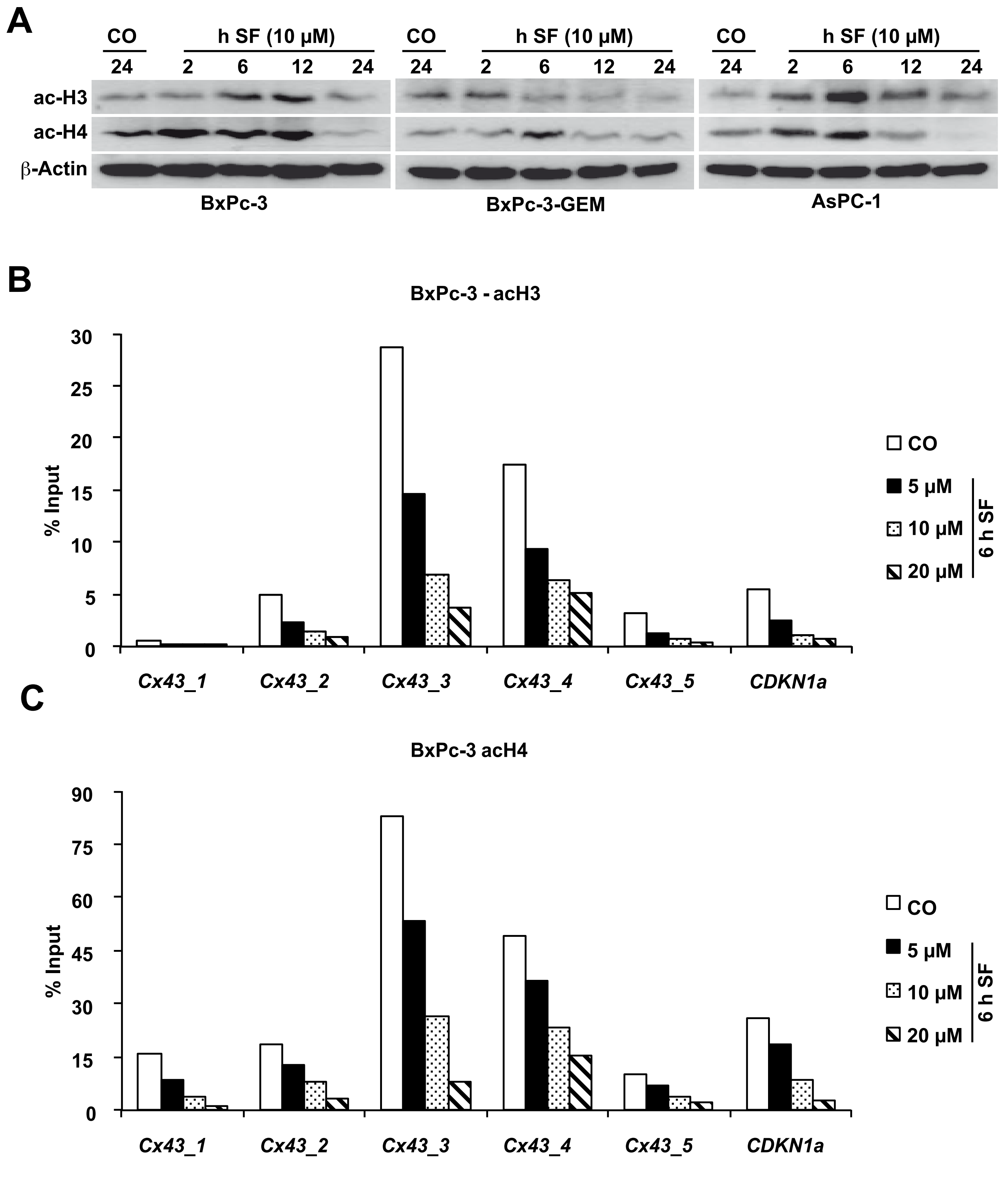

Forster et al., Supplemental Figure 7
